# Improving Corrosion Resistance of Zircaloy-4 via High-Current Pulsed Electron Beam Surface Irradiation

**DOI:** 10.3390/ma18010076

**Published:** 2024-12-27

**Authors:** Shen Yang, Heran Yao, Zhiyong Hu, Tao Chen

**Affiliations:** Ministry of Education Key Laboratory for Cross-Scale Micro and Nano Manufacturing, College of Mechanical and Electrical Engineering, Changchun University of Science and Technology, Changchun 130022, China; yhr202411@163.com (H.Y.); hzy14798@126.com (Z.H.); jsssys2024@163.com (T.C.)

**Keywords:** high current pulsed electron beam (HCPEB), Zircaloy-4, microstructure, corrosion resistance

## Abstract

Zircaloy-4 is extensively used in nuclear reactors as fuel element cladding and core structural material. However, the safety concerns post-Fukushima underscore the need for further enhancing its high-temperature and high-pressure water-side corrosion resistance. Therefore, this study aimed to investigate the effects of high-current pulsed electron beam (HCPEB) irradiation on the microstructures and corrosion resistance of Zircaloy-4, with the goal of improving its performance in nuclear applications. Results showed that after irradiation, the cross-section of the sample could be divided into three distinct layers: the outermost melted layer (approximately 4.80 μm), the intermediate heat-affected zone, and the bottom normal matrix. Large numbers of twin martensites were induced within the melted layer, which became finer with increasing irradiation times. Additionally, plenty of ultrafine/nanoscale grains were observed on the surface of the sample pulsed 25 times. Zr(Fe, Cr)_2_ second-phase particles (SPPs) were dissolved throughout the modified layer and Fe and Cr elements were uniformly distributed under the action of HCPEB. As a result, the corrosion resistance of the sample pulsed 25 times was significantly improved compared to the initial one. Research results confirmed that HCPEB irradiation is an effective method in improving the service life of Zircaloy-4 under extreme environmental conditions.

## 1. Introduction

With the increasing scarcity of fossil fuels, nuclear energy that has the advantages of high efficiency, low carbon, and cleanliness has attracted widespread attention and become one of the most promising energy sources [1,2]. However, due to certain risks when using nuclear energy, nuclear safety has become an important issue around the globe [3]. Zircaloy-4, a zirconium-based alloy, possesses a unique combination of properties, such as a low neutron absorption cross-section, good high-temperature strength, and excellent high-temperature and high-pressure water-side corrosion resistance, making it ideal for use in nuclear reactors, particularly as cladding material in fuel assemblies. Its critical role in ensuring the performance, safety, and longevity of nuclear reactors cannot be overstated [4,5]. While the Fukushima nuclear accident was caused, at least in part, by the reacted between zirconium alloy and high-temperature steam, which released a large amount of hydrogen gas that triggered a hydrogen explosion, leading to a series of serious consequences. Therefore, it is important to explore effective ways to improve the corrosion resistance of Zircaloy-4 against high-temperature and high-pressure water-side corrosion to further promote the safety of nuclear reactors.

At present, researchers mainly improve the corrosion resistance of zirconium alloys through the following approaches: developing new types of zirconium alloys [6,7], adjusting thermal processing techniques [8,9], and surface modification of existing zirconium alloys [10,11,12,13,14,15,16]. Compared to the other two ways, surface modification methods have garnered the most discussion and research among scholars as they do not affect the comprehensive performance of the alloy matrix and have various methods. Among the surface modification methods for zirconium alloys, the most common is surface coating technology [10,11], followed by laser surface treatment [12], ion implantation [13,14,15], ion irradiation [16], etc. However, each method inevitably has certain issues. For instance, surface coatings will increase the material’s thickness and reduce the thermal conductivity of zirconium alloys, and there are concerns about the adhesion between the coating and the matrix; laser treatment technology has issues such as a small beam spot, low effective energy utilization, and susceptibility to oxidation of the modified surface; and ion beams have limitations such as limited penetration depth and the high cost of equipment.

In recent years, a high-current pulsed electron beam (HCPEB) surface modification technology with the advantages of high energy utilization, large depth of penetration, low cost, high efficiency, simplicity, and reliability has been gradually developed and has attracted more and more scholars’ attention. Its main characteristic is that under vacuum conditions, the accelerating voltage accelerates the electrons emitted by the electron gun and converges them into a high-energy electron beam (with an energy density up to 10^8^–10^9^ W/cm^2^). Within a few microseconds or even nanoseconds, this beam penetrates the material surface, causing rapid heating (at a rate up to 10^9^ K/s), melting, or even vaporization. Subsequently, it relies on the heat conduction of the substrate itself to cool down (at a rate up to 10^7^ K/s) and solidify (at speeds of 2–10 m/s). At the same time, the extremely high temperature gradient on the material surface (up to 10^8^ K/m) also induces high-magnitude thermal stresses (up to hundreds of MPa-GPa). Under the combined action of the non-equilibrium temperature field and the dynamic thermal stress field, the material surface can achieve special modification effects that are difficult to achieve with conventional surface modification methods, such as purification and homogenization of the sample surface, formation of a large number of fine crystals, supersaturated solid solutions, crystal defects, deformed structures, etc. [17,18,19,20,21,22]. A large number of previous studies have shown that HCPEB technology is an effective method to improve the surface corrosion resistance of various materials [23,24,25,26,27,28,29,30,31,32,33,34].

In view of this, the authors also attempted to use HCPEB to modify the surface of Zircaloy-4 in a previous work [35]. However, due to the limitation of the test conditions at that time, only the electrochemical workstation was used to test the corrosion resistance of the samples before and after modification at room temperature. Considering the actual service conditions of Zircaloy-4, in the present work, the corrosion resistance of the sample after HCPEB modification was investigated under 500 °C/10.3 MPa superheated steam, and the microstructures and corrosion behavior after irradiation will be discussed in detail by means of more observation and analysis with a transmission electron microscope (TEM). The research results will provide the necessary theoretical and experimental data for the study of improving the service life of Zircaloy-4 under extreme environmental conditions through HCPEB surface modification.

## 2. Materials and Methods

An annealed Zircaloy-4 plate (Sn 1.38, Fe 0.22, Cr 0.12, Zr balance, wt%) was used in this study. Initially, the plate was cut into numerous 10 mm × 10 mm × 6 mm samples. Then, one of the 10 × 10 mm surfaces of each sample was sanded and polished for use. Subsequently, the irradiation treatment was carried out on the polished surfaces using the “HOPE-I”-type HCPEB equipment. More details about the principles of the HCPEB equipment are in references [20,22]. The irradiation parameters used are shown in Table 1. A corrosion test of samples before and after surface modification was examined by using a high-temperature and high-pressure static reactor in the corrosion environment of 500 °C/10.3 MPa superheated steam, and the corrosion time was 24 h. After surface modification and corrosion test, X-ray diffractometry (XRD, Rigaku D/max-2500/pc, Tokyo, Japan, CuKα, scan speed: 4°/min) was employed to examine the surface phase structures of the samples, a scanning electron microscope (SEM, JEOL, JSM-7800 F, Tokyo, Japan) was utilized to analyze the surface and cross-section morphologies of the samples, a transmission electron microscope (TEM, JEOL, JEM-2100 F, Tokyo, Japan, equipped with a scanning TEM (STEM) attachment and an energy dispersive spectrometer (EDS)) was adopted to study the internal structures within the modified layers and the oxide films. The TEM samples of the oxide films were prepared by focused ion beam (FIB).

## 3. Results

### 3.1. XRD Analysis

**Figure 1:**
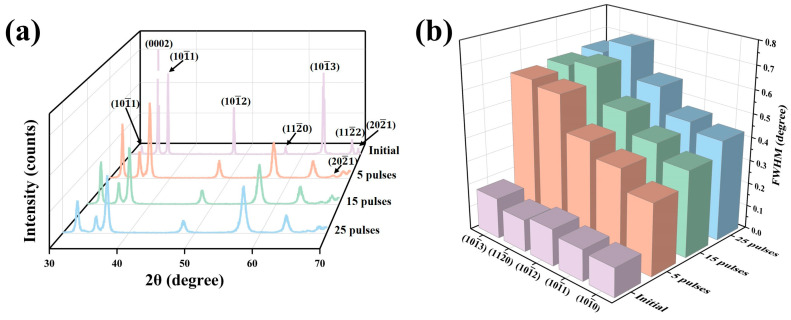
(**a**) XRD patterns of the sample surfaces before and after HCPEB irradiation, (**b**) change regularities of the FWHM for partial crystal planes before and after HCPEB irradiation.

Figure 1a shows the XRD patterns of the sample surfaces before and after HCPEB irradiation. During the process of HCPEB irradiation, the instantaneous high temperature melted the surface layer. However, due to the extremely short melting and cooling time, the rapid cooling process induced a martensitic transformation, forming a large number of α′ martensites [36]. Because the α′-Zr phase has the same crystal structure as the α-Zr phase, it could not be accurately identified here and will be further explored in the following text [37,38,39]. Figure 1b shows the change regularities of the full width at half maximum (FWHM) for partial crystal planes before and after HCPEB irradiation. It can be clearly seen that compared with the initial sample, the XRD diffraction peaks of the irradiated samples were significantly broadened, and the FWHM increased gradually with the increase in irradiation times, indicating that the substructures within the surface layer were refined continuously under the action of the HCPEB irradiation [40,41,42].

### 3.2. Microstructural Analysis

**Figure 2:**
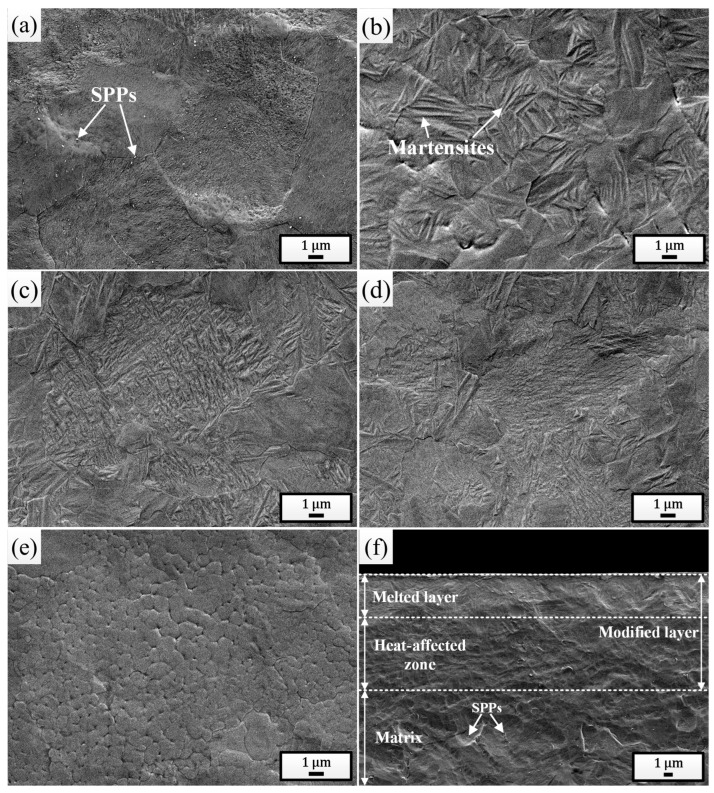
Surface SEM morphologies of the initial sample (**a**) and the samples pulsed 5 times (**b**), 15 times (**c**), and 25 times (**d**,**e**). (**f**) Cross-sectional SEM morphology of the sample pulsed 25 times.

Figure 2a shows the surface SEM morphology of the initial sample. Prior to observation, the polished surface was etched using a metallographic corrosion solution consisting of 10% HF + 45% HNO_3_ + 45% H_2_O (volume fraction). It can be clearly seen that the sample surface was composed of α-Zr grains with a large number of Zr(Fe, Cr)_2_ secondary phase particles (SPPs) distributed within them and along the grain boundaries, and other microstructures were not found. Figure 2b shows the surface SEM morphology of the sample pulsed 5 times. It is evident that a significant amount of martensites were formed on the irradiated surface with an average width of 0.11 μm, confirming the occurrence of martensitic phase transformation. With increase in irradiation times (Figure 2c–d), the martensites became finer and finer [43,44]. After irradiation with 25 pulses, the average width of martensites was as low as 0.02 μm. Additionally, large numbers of ultrafine grains were also observed on the surface of the sample pulsed 25 times, with an average size of 0.49 μm, as shown in Figure 2e. This is due to the increased heat accumulation in the surface layer, which extended the melting time to some extent, promoting the nucleation of local primary grains, whereas limited cooling time prevented these grains from growing larger, ultimately returning to room temperature [45]. It can also be observed that martensites were rarely found within these ultrafine grains, as the martensitic transformation would be inhibited when the grain size was sufficiently small [46]. Figure 2f provides the cross-sectional SEM morphology of the sample pulsed 25 times. It can be seen clearly that the cross-section was composed of a distinct three-layer structure: the outermost white, bright melted layer (approximately 4.80 μm), the intermediate heat-affected zone, and the bottom normal matrix. It is also noticeable that the SPPs were almost absent in both the melted layer and the heat-affected zone, indicating that under the action of HCPEB, the Zr(Fe, Cr)_2_ SPPs were significantly dissolved. Especially for the heat-affected zone, although the heat conducted to this layer was not sufficient to melt the matrix, it was enough to cause the dissolution of the SPPs.

**Figure 3:**
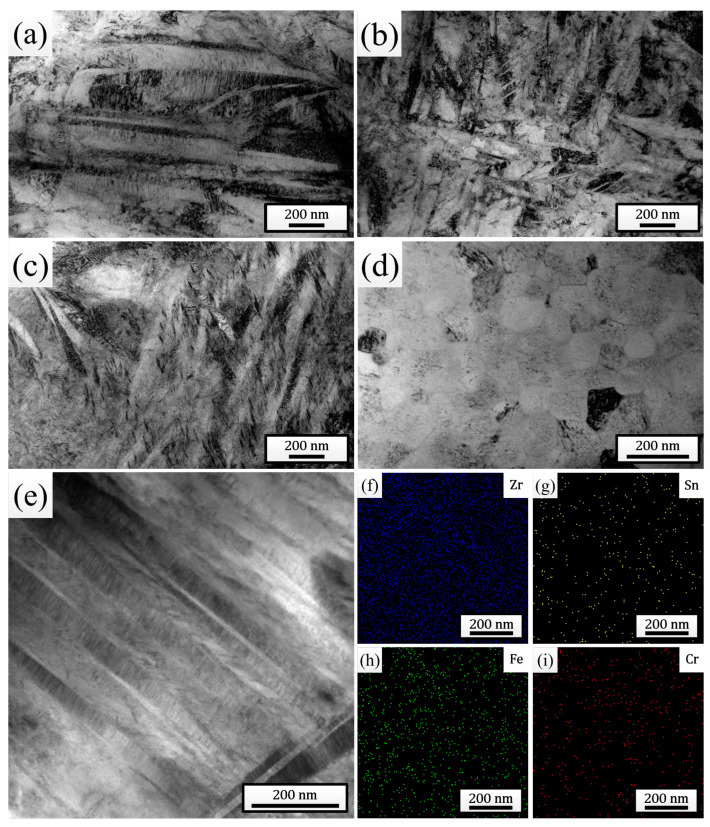
TEM bright-field morphologies of the melted layers after irradiation with 5 pulses (**a**), 15 pulses (**b**), and 25 pulses (**c**,**d**). (**e**) STEM bright-field morphology of the melted layer after 25 pulses of HCPEB irradiation. (**f**–**i**) EDS mapping results of elements in (**e**).

Figure 3a–d show the TEM bright-field morphologies of the melted layers after different times of HCPEB irradiation. The observation results were completely consistent with the XRD and SEM analysis results, both clearly indicating the dissolution of the SPPs and the continuous refinement of the martensites after irradiation. For the sample pulsed 25 times, a large number of nanoscale martensites with widths even lower than 10 nm were distinctly observed (Figure 3c). In addition, plenty of twin substructures were found within the martensitic plates, indicating that the martensitic type within the melted layer was mainly twin martensite. Additionally, for the sample pulsed 25 times, plenty of nanoscale grains with sizes less than 100 nm (further refined compared to that observed in Figure 2e, with an average size of 70.50 nm) were also observed (Figure 3d), within which martensites indeed could not be found, confirming the point in the previous paragraph. To study the distribution of elements inside the melted layer, a local area of the sample pulsed 25 times was selected for EDS mapping analysis (Figure 3e), and the results are shown in Figure 3f–i. It can be clearly seen that all the elements dissolved in the matrix were distributed uniformly, and the phenomenon of local evident aggregation did not occur, indicating that HCPEB irradiation promoted the uniform distribution of elements within the sample surface.

### 3.3. Corrosion Behavior

**Figure 4:**
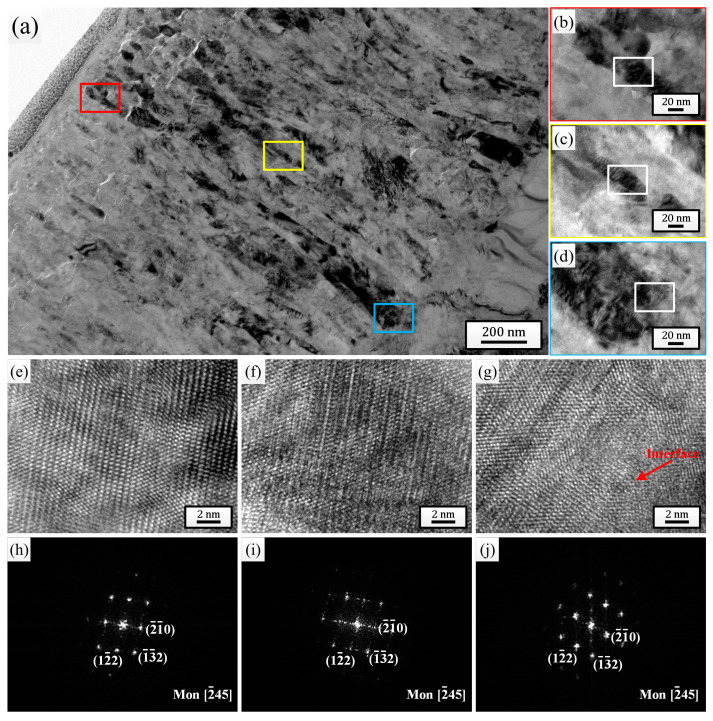
Cross-sectional TEM bright field morphology of the oxide film grown on the initial sample after 24 h exposure to 500 °C/10.3 MPa superheated steam. (**b**–**d**) High-magnification images of the regions marked with red, yellow and blue squares in (**a**), respectively. (**e**–**g**) HRTEM images of the regions marked with white squares in (**b**–**d**), respectively. (**h**–**j**) Corresponding FFT patterns of (**e**–**g**), respectively.

**Figure 5:**
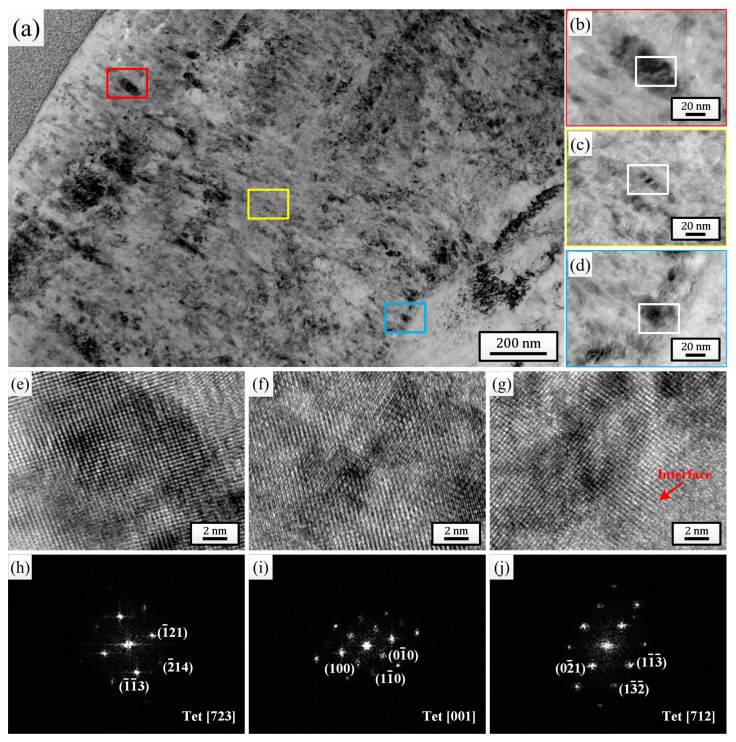
Cross-sectional TEM bright field morphology of the oxide film grown on sample pulsed 25 times after 24 h exposure to 500 °C/10.3 MPa superheated steam. (**b**–**d**) High-magnification images of the regions marked with red, yellow and blue squares in (**a**), respectively. (**e**–**g**) HRTEM images of the regions marked with white squares in (**b**–**d**), respectively. (**h**–**j**) Corresponding FFT patterns of (**e**–**g**), respectively.

For the comprehensive microstructure analysis results, the sample pulsed 25 times was chosen to investigate the effect of HCPEB irradiation on the corrosion behavior of Zircaloy-4. After 24 h exposure to 500 °C/10.3 MPa superheated steam, the cross-sectional TEM bright-field morphologies of the oxide films grown on the initial sample (Figure 4) and the sample pulsed 25 times (Figure 5) were observed and analyzed. By comparing Figure 4a and Figure 5a, it can be observed that the thickness of the oxide film grown on the sample pulsed 25 times (approximately 1.57 μm) was evidently lower than that grown on the initial one (approximately 1.88 μm). And after only 24 h of corrosion, the outermost layers of both oxide films exhibited significant loose areas, characterized by the presence of many small-sized micro-pores and horizontal micro-cracks. But, it is evident that the thickness of the loose area for the sample pulsed 25 times was significantly lower than that of the initial one, and the size and quantity of micro-pores and micro-cracks within this area were also much lower. Of course, apart from the outermost layer, a small number of micro-pores and micro-cracks could also be observed in other areas of the oxide films for both samples, but without a doubt, the oxide film of the sample pulsed 25 times displayed a better compactness in every area. Figure 4b–d and Figure 5b–d are high-magnification images of the regions marked with red (outer layer), yellow (middle layer), and blue (bottom layer) squares corresponding to each figure, respectively. It is well known that the ZrO_2_ growing on the Zr matrix can mainly be divided into two types: m-ZrO_2_ and t-ZrO_2_. The t-ZrO_2_ phase possesses good chemical stability and low lattice mismatch, which can enhance the adhesion and crack resistance of the oxide film. This contributes to the formation of a uniform and compact oxide film, thereby enhancing the corrosion resistance of the zirconium alloys. In contrast, m-ZrO_2_ may lead to the embrittlement of the oxide film and the formation of cracks, resulting in a less compact oxide film and reducing the corrosion resistance of the zirconium alloys [47]. To test the phase composition of the ZrO_2_ grains at different oxide film depths for the two samples, high-resolution observation was performed on the regions marked with white squares in Figure 4b–d and Figure 5b–d, and the high-resolution TEM (HRTEM) images and their corresponding fast Fourier transform (FFT) images are shown in Figure 4e–j and Figure 5e–j, respectively. It can be seen that for the initial sample, the grains selected at the outer layer, middle layer, and oxide/matrix (O/M) interface were all m-ZrO_2_ phase, while for the sample pulsed 25 times, the grains selected were all the t-ZrO_2_ phase. This result further confirmed that the sample pulsed 25 times had better high-temperature and high-pressure corrosion resistance. Moreover, the dispersively distributed white, bright lines between the diffraction spots in Figure 4h–j could intuitively indicate that there existed many stacking faults in the selected grains, while almost no bright lines are observed between the diffraction spots in Figure 5h–j, indicating that there existed fewer defects inside the grains.

## 4. Discussion

The Pilling–Bedworth ratio (P.B. ratio, the ratio of the volume of the metal oxide to the volume of the metal consumed in forming that oxide) of ZrO_2_/Zr is 1.56, which means that the volume of the oxide film will expand during the oxidation process. Due to the constraint of the Zr matrix, significant compressive stress will be generated within the oxide film [48]. Consequently, the formation of ZrO_2_ is accompanied by the generation of defects such as vacancies, dislocations, interstitial atoms, etc. Under the combined effects of high temperature, compressive stress, and time, these defects will migrate and coalesce, promoting the formation of micro-pores and micro-cracks. At the same time, this process will also accelerate the transformation of the t-ZrO_2_ phase to the m-ZrO_2_ phase [47,49]. In other words, the protective characteristics of the oxide film will gradually weaken.

Research indicated that the presence of higher concentrations of alloying elements Fe and Cr in the oxide film could inhibit the formation of micro-pores and micro-cracks in ZrO_2_ grains by reducing the stress concentration in the oxide film, stabilizing the t-ZrO_2_ phase, enhancing the adhesion between the oxide film and the matrix, hindering the grain boundary migration, altering the growth dynamics of the oxide film, etc. [9,50]. Obviously, during the corrosion process, alloying elements dissolved in the Zr matrix were more likely to dissolve into the oxide film than those in the SPPs. That is to say, due to the substantial dissolution of SPPs in the Zr matrix, the concentration of alloying elements in the oxide film grown on the HCPEB irradiated sample was significantly higher than that grown on the initial sample. At the same time, the distribution of alloying elements in the matrix after repeated remelting was more uniform, which undoubtedly played a better role in enhancing the corrosion resistance of the alloy. In contrast, the oxidation of SPPs in the matrix would generate local additional stresses in the oxide film, accelerating the process of corrosion [6].

Additionally, after multiple irradiations, a high-volume fraction of boundaries was provided by large numbers of ultrafine/nanoscale grains, martensites, and twins. These boundaries collectively served as rapid diffusion pathways for Fe and Cr atoms to the sample surface, playing an extremely important role in the formation of a dense protective oxide film.

Research results provide a new method for the surface modification of Zircaloy-4 to improve its service life in nuclear reactors and also provide a new idea for the modification research of other zirconium alloys and even other alloys in industrial applications. Of course, the applicability and effectiveness of HCPEB technology may vary when dealing with different materials, which requires the adjustment and optimization of process parameters according to the characteristics of different materials. At the same time, the long-term stability and reliability of HCPEB technology in industrial applications also need to be ensured through testing and verification in actual production.

## 5. Conclusions

HCPEB surface treatment was applied to Zircaloy-4 with varying times of irradiation. During irradiation, the sample surface was melted, within which martensitic phase transformation occurred, resulting in the formation of large numbers of twin martensites. With the increasing of irradiation times, these martensites became finer and finer. After 25 pulses of irradiation, the average width of the martensites was as low as 0.02 μm, and plenty of ultrafine/nanoscale grains were also observed. Zr(Fe, Cr)_2_ SPPs were dissolved both in the melted layer and the heat-affected zone, and the alloying elements Fe and Cr were uniformly distributed under the action of HCPEB irradiation. After 24 h corrosion in 500 °C/10.3 MPa superheated steam, the thickness of the oxide film grown on the sample pulsed 25 times was reduced by 19.75% compared to that grown on the initial one, and the oxide film was significantly improved in uniformity and compactness.

The research results provide necessary theoretical and experimental data for the study of improving the service life of Zircaloy-4 under extreme environmental conditions through HCPEB surface modification. In future work, more characterization methods will be employed for a detailed analysis of the modified surface, and corrosion tests will be conducted over extended exposure times to provide further data support for this research.

## Figures and Tables

**Table 1:** HCPEB irradiation parameters.

Accelerating Voltage	Pulse Duration	Energy Density	Vacuum	Number of Irradiation Times
27 kV	1.5 μs	4 J/cm^2^	5 × 10^−3^ Pa	5, 15, 25

## Data Availability

All research data supporting this publication are directly available within this publication.
